# Anticandidal Activity of Kalopanaxsaponin A: Effect on Proliferation, Cell Morphology, and Key Virulence Attributes of *Candida albicans*

**DOI:** 10.3389/fmicb.2019.02844

**Published:** 2019-12-03

**Authors:** Ying Li, Mingzhu Shan, Mingju Yan, Huankai Yao, Yuechen Wang, Bing Gu, Zuobin Zhu, Hongchun Li

**Affiliations:** ^1^School of Medical Technology, Xuzhou Medical University, Xuzhou, China; ^2^Jiangsu Key Laboratory of New Drug Research and Clinical Pharmacy, School of Clinical Pharmacy, Xuzhou Medical University, Xuzhou, China; ^3^Department of Genetics, Xuzhou Medical University, Xuzhou, China; ^4^Department of Laboratory Medicine, Affiliated Hospital of Xuzhou Medical University, Xuzhou, China

**Keywords:** Kalopanaxsaponin A, *Candida albicans*, virulence factors, hyphae, farnesol, cAMP

## Abstract

**Background:**

The pathogenicity of *Candida albicans* is attributed to various virulence factors including adhesion to the surface of epithelial cells or mucosa, germ tube formation, hyphal morphogenesis, development of drug resistant biofilms, and so on. The objective of this study was to investigate the effects of Kalopanaxsaponin A (KPA) on the virulence of *C. albicans*.

**Methods:**

The effect of KPA on the virulence of *C. albicans* was characterized by an XTT reduction assay and fluorescent microscopic observation. The action mechanism was further explored using GC/MS system and BioTek Synergy2 spectrofluorophotometry. The cytotoxicity and therapeutic effect of KPA were evaluated by the *Caenorhabditis elegans*-*C. albicans* infection model *in vivo*.

**Results:**

The minimum inhibitory concentration (MIC) of KPA was 8∼16 μg/mL for various genotypes of *C. albicans*. The compound was identified as having remarkable effect on the adhesion, morphological transition and biofilm formation of *C. albicans*. The results of fluorescent microscopy and GC/MS system suggested that KPA could promote the secretion of farnesol by regulating the expression of Dpp3 and decrease the intracellular cAMP level, which together inhibited morphological transition and biofilm formation. Notably, KPA showed low toxicity *in vivo* and a low possibility of developing resistance.

**Conclusion:**

Our results demonstrated that KPA had remarkable efficacy against *C. albicans* pathogenicity, suggesting that it could be a potential option for the clinical treatment of candidiasis.

## Introduction

*Candida albicans* is a component of the normal flora of healthy human beings, residing on mucosal surfaces and in the gastrointestinal and genitourinary tracts (Ganguly and Mitchell, 2011). However, when the anatomical barrier is broken or the host immune function is disturbed, *C. albicans* can cause skin and mucous membrane infection or life-threatening systemic infection (Mayer et al., 2013; Hube et al., 2015). In the United States, *C. albicans* is the fourth most common microbe causing blood infections with a crude mortality rate of about 50% (Haynes, 2002; Pfaller and Diekema, 2007). With the increasing severe situation of *C. albicans* infection, antifungal agents have been developed and widely used in the treatment of various *C. albicans* infections. However, resistance has become a challenge in the clinical treatment of *C. albicans* infections due to wide use or even abuse of antifungal drugs in the recent decade. It is therefore an urgent task to develop new drugs against *C. albicans* resistant infections (Haque et al., 2016).

The pathogenicity of *C. albicans* depends on various virulence factors, such as adhesion proteins, morphological transformation, secreted aspartyl proteases and phospholipases, phenotypic switching and biofilm formation (Sudbery et al., 2004). There exist at least three morphologies for this organism: a yeast budding form, pseudohyphae, and a filamentous form. *C. albicans* cells initially adhere to host cells in the yeast budding form, which can grow normally on the mucosal and skin surface of the host without causing immune response. When the growing environment is suitable for hyphal formation, the yeast cells transform to hyphae, which has intensive ability of tissue invasion and infiltration, playing an important role in the pathogenesis (Ramage et al., 2005). Hyphal formation can promote the biofilm formation of *C. albicans*. Mature biofilms of *C. albicans* have complex structures consisting of pseudohyphae, hyphae and yeast cells surrounded by exopolymeric matrices, which provide a protective barrier against antifungal therapy, escape cells from the immune system and provide a source of infection for infectious relapse (Bonhomme and d’Enfert, 2013). Thus, inhibiting *C. albicans* virulence has be recognized as a promising strategy for the treatment of fungal infections as comparing with traditional antifungal drugs targeting cell growth (Biswas et al., 2007).

**FIGURE 1:**
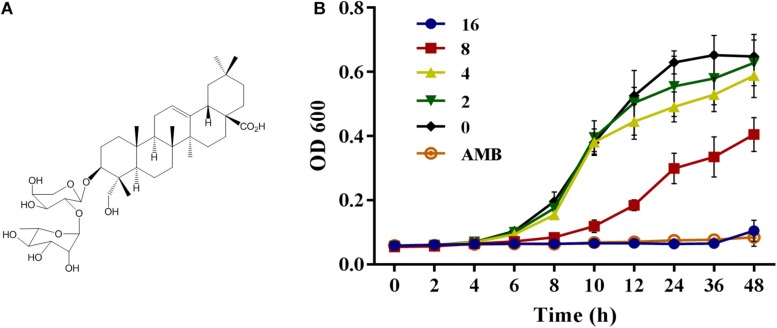
**(A)** The structure of Kalopanaxsaponin A (KPA). **(B)** The growth curve of wild type *C. albicans* strain YEM30 under KPA treatment. YEM30 cells were diluted in the YPD broth and incubated with different doses of KPA at 30°C. OD600 of each group was detected with a Bio-Rad microplate reader every 1 h. Results were shown as means ± SDs.

Natural products have long been an important source of new drug discovery. From 1981 to 2014, nearly 50% new drugs approved by the United States Food and Drug Administration were either natural product or based thereon (Newman and Cragg, 2012). Kalopanaxsaponin A (KPA) is a triterpenoid saponin isolated from the stem bark of *Kalopanax pictus* in our laboratory (Figure 1A). Previous studies have showed that KPA has various bioactivities, such as antifungal (Lee et al., 2001), anti-inflammatory (Kim Y.K. et al., 2002; Jeong et al., 2013), anti-rheumatic (Kim D.H. et al., 2002), anti-tumor (Park et al., 2001) and anti-diabetes (Kim et al., 1998) activities. It has also proved to improve memory deficits by inhibiting the acetylcholinesterase activity (Joh et al., 2012). In this study, we found that KPA could inhibit the proliferation of yeast cells and reduce the virulence of *C. albicans* by inhibiting adhesion, hyphae and biofilm formation. Further exploration showed that this inhibitory effect of KPA was attributed to the decrease content of intracellular cyclic adenosine monophosphate (cAMP) and the secretion of farnesol induced by Dpp3 expression. In addition, KPA could prolong the survival time of infected *Caenorhabditis elegans* in a *C. elegans*-*C. albicans* infectious model. These finding suggest that KPA may be a promising candidate for use to target fungal virulence against clinically relevant fungal infections.

## Materials and Methods

### Strains

Two wild-type strains (SC5314 and YEM30), two azole-resistant strains (CA10 and CA148) (Shandong Province Qianfoshan Hospital, China) (Sun et al., 2009), two mutant strains (DSY448 and DSY1050), and a type culture strain (ATCC10231, Manassas, VA, United States) of *C. albicans* were used in this study. Wild type *C. elegans* strain N2 was obtained from the Caenorhabditis Genetics Center, United States. All strains were stored in the medium containing 20% glycerol at −80°C for a long time. Prior the experiment, *C. albicans* was inoculated twice on YPD solid plates (yeast extract 1%, peptone 2%, glucose 2%, and agar 2%) and cultured at 30°C. The grown single colonies were inoculated into the liquid YPD broth (2% tryptone, 1% yeast extract, and 2% glucose) and cultured overnight at 30°C, 200 rpm.

### Chemicals

Kalopanaxsaponin A was a separated compound extracted from the stem of the *Kalopanax* in our laboratory, with a purity over 98% as analyzed by high-performance liquid chromatography. KPA (10 mg/mL) and amphotericin B (AMB, Sigma, 10 mg/mL) were dissolved in dimethyl sulfoxide (DMSO) (Sigma, St. Louis, MO, United States) to a stock solution and frozen at −20°C until use. In each assay, the content of DMSO was below 1%.

### Minimum Inhibitory Concentration (MIC) Test

The MIC values of KPA against various genotypes *C. albicans* were detected by the broth microdilution method as previously described (M27-E4) (Li et al., 2019).

### Proliferation Inhibition Test

The YEM30 strains were diluted to an initial concentration of 1 × 10^6^ cells/mL with YPD broth. Add KPA to a final concentration of 0, 2, 4, 8, and 16 μg/mL, and transferred l to 96-well flat-bottom plates at 100 μL/well. 2 μg/mL of AMB and KPA-free group were served as positive and negative control, respectively. After incubation at 37°C, the absorbance of 600 nm was detected every 2 h with a Bio-Rad Model 680 microplate reader (Bio-Rad Laboratories, Richmond, CA, United States).

### Adhesion Assay

The effect of KPA on *C. albicans* adhesion was evaluated by the XTT reduction assay as previously described (Li et al., 2019), using AMB (2 μg/mL) as positive control. After culture and wash, treated YEM30 cells were observed directly under a microscope (Olympus IX71, Olympus, Tokyo, Japan) in the bright field mode. Meanwhile, the remaining adherent cells on the bottom were detected by the XTT reduction assay using an XTT Cell Proliferation Assay Kit (BestBio, Shanghai, China).

### Morphological Transition Test

A YEM30 cell suspension (1 × 10^5^ cells/mL) was prepared in RPMI 1640 containing different concentrations of KPA (0∼16 μg/mL) and incubated at 37°C without shaking. The cell morphology was monitored by microscopy (Olympus IX71, Olympus, Tokyo, Japan) every 4 h.

### Effect of KPA on *C. albicans* Biofilm Formation

The effect of KPA on biofilm formation was detected in 96-well flat-bottom plates using the XTT reduction assay as previously described (Li et al., 2019). YEM30 cells (1 × 10^6^ cells/mL in RPMI 1640) were seeded into a 96-well plate with final concentrations of 0, 4, 8, 16, and 32 μg/mL of KPA. The plate was incubated for 24 h at 37°C in static culture. The supernatant was removed by three washes with sterile PBS. The XTT reduction assay was performed using XTT Cell Proliferation Assay Kit (BestBio, Shanghai, China), biofilm formation was observed with an Olympus microscope in the bright field mode.

### The cAMP Rescue Test

To determine the effect of cAMP on the filamentation under KPA treatment, YEM30 cells were prepared as for the above morphological transition test. After the addition of 0, 12 μg/mL KPA, dibutyryl-cAMP (db-cAMP, Sigma) was added with a final concentration of 1 mM. The db-cAMP free groups served as control. The cell morphology was monitored by Olympus microscope every 4 h.

*Candida albicans* cells (1 × 10^5^ cells/mL) were diluted by RPMI 1640 medium with 16 μg/mL KPA, followed by the addition of db-cAMP at a final concentration of 1 mM. The db-cAMP-free cells with or without KPA served as a control. After 24-hour incubation at 37°C, the medium was discarded, and each well was washed three times with PBS to remove the non-adhered cells. The formed biofilms were imaged microscopically and measured using the XTT reduction assay.

### Quantification of Farnesol Secreted in the Supernatant

YEM30 cells were diluted to 1 × 10^5^ cells/mL with RPMI 1640 medium containing different concentrations of KPA. After 12-hour cultured at 37°C, *C. albicans* cells and culture supernatants were collected. Dry weight of *C. albicans* cells was weighted. Farnesol was extracted with the same volume ethyl acetate of supernatants, and the content was quantified using an Agilent model 5975C/7697A GC/MS system as previously described (Li et al., 2015). The content of farnesol was calculated by the standard curve and cell dry weight.

### Measurement of Dpp3 Expression

The expression of Dpp3 in BWP17-*DPP3*-*GFP* strain was detected after KPA treatment (Zhang et al., 2011). Cells (about 1 × 10^5^ cells/mL) treated by different concentrations of KPA were cultured in RPMI1640 medium at 37°C for 3 h. Changes of Dpp3-gfp expression were observed using an Olympus BX53F fluorescence microscope and measured with a BioTek Synergy2 spectrofluorophotometer at 486 nm excitation and 528 nm emission wavelengths.

### Toxicity Evaluation

To evaluate the toxicity of KPA, the survival rate of wild type *C. elegans* was with monitored after KPA treatment. The effect of KPA on the survival of wild type *C. elegans* N2 was detected as previously described (Chang et al., 2012). Briefly, the prepared nematodes treated with KPA were incubated in a 96-well plate at 25°C for 2 days. The survival and death rates were monitored and calculated by counting the numbers of treated worms under the microscope.

### *C. elegans*-*C. albicans* Infection Model

*C. elegans-C. albicans* infectious model was used to evaluate the antifungal activity of KPA *in vivo* as previously reported (Li et al., 2015). Briefly, worms were exposed to *C. albicans* strain YEM30 for 2 h and cultivated with different concentrations of KPA at 25°C. The survival state of infected worms was monitored daily to calculate survival rates.

### Statistical Analysis

All results were expressed as the mean of the corresponding standard deviation (SD) of three measurements from three independent experiments. The log rank test was used to analyze the data of *C. elegans*-*C. albicans* infection assay. The other experimental data were statistically analyzed using Student’s *t*-test (two-tailed, unequal variance). *P* < 0.05 was considered statistically significant.

## Results

### Effect of KPA on Viability of *C. albicans* Cells

To evaluate the effect of KPA on *C. albicans* proliferation, the MIC value of KPA against *C. albicans* of different genotypes was determined *in vitro*. It was found that KPA had effective antifungal activity against various genotypes *C. albicans*. As shown in Table 1, the MIC values of all tested strains were 8∼16 μg/mL (KPA) and 0.5∼1 μg/mL (AMB, positive control), respectively.

**TABLE 1:** MIC of KPA and AMB against different genotypes of *C. albicans in vitro.*

**Strains^†^**	**MIC of drugs (μg/mL)^‡^**	
	**KPA**	**AMB**
ATCC10231	16	0.5
SC5314	16	0.5
YEM30	16	0.5
DSY448	16	0.5
DSY1050	16	0.5
CA10	16	1
CA148	16	0.5

*^†^ATCC10231, SC5314 and YEM30 are wild-type strains. The genotype of DSY448 is Δ*cdr1*:*hisG-URA3-hisG*/Δ*cdr1*:*hisG*; The genotype of DSY1050 is Δ*cdr1*:*hisG*/Δ*cdr1*:*hisG* Δ*cdr2*:*hisG*/Δ*cdr2*:*hisG* Δ*mdr1*:*hisG-URA3-hisG*/Δ *mdr1*:*hisG*. CA10 and CA148 are clinical isolated azoles multi-resistance *C. albicans* isolates. ^‡^KPA, Kalopanaxsaponin A; AMB, amphotericin B.*

Besides detection of MIC value, the inhibitory proliferation curve of KPA was also plotted (Figure 1B). Compared with the negative control group, 1/2 MIC (8 μg/mL) of KPA showed an inhibitory effect on the proliferation of *C. albicans*. The MIC (16 μg/mL) totally inhibited cell growth to a level almost equal to the positive control (AMB, 2 μg/mL). In conclusion, KPA had significant antifungal activity against *C. albicans*.

### Effect of KPA on the Adhesion Ability of *C. albicans*

**FIGURE 2:**
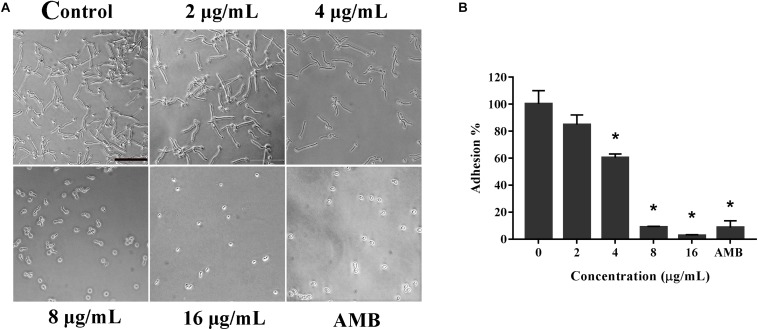
The effect of KPA on the adhesion of *C. albicans.* The effect of TC on the adhesion of *C. albicans* was detected by the microscopy **(A)** and XTT reduction assay **(B)**. The bar in panel **(A)** indicates 50 μm. Results in panel **(B)** are showed as means ± SDs. Asterisks (^∗^) represent significance with *P* < 0.01.

Knowing that the adhesion ability is one of the primary independent factors contributing to the virulence of *C*. *albicans*, we detected the effect of KPA on the adhesion of *C. albicans* at the bottom of the 96-well plates (Sudbery et al., 2004). Microscopically, as shown in Figure 2A, the number of adherent cells under 4, 8, and 16 μg/mL of KPA was smaller than that of the drug-free group. The same trend was observed in the XTT assay data, showing that 2 and 4 μg/mL KPA prevented about 10 and 40% cells from adhering to the substratum of the plate, respectively. The inhibition rate increased to 90% or nearly 100% when the concentration was increased to 8 or 16 μg/mL (*P* < 0.05). The inhibition rate in the AMB positive control group was 95% (Figure 2B). To sum up, KPA inhibited *C. albicans* adherence in a dose-dependent manner.

### KPA Inhibited the Yeast-to-Hyphal Transition of *C. albicans*

**FIGURE 3:**
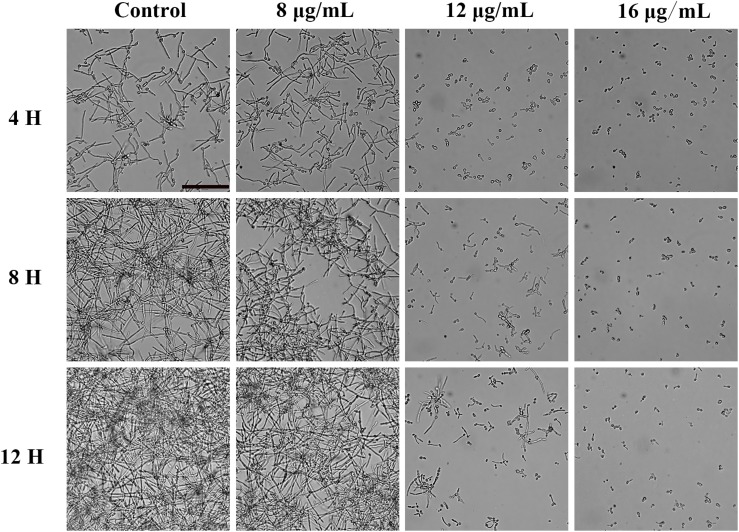
The effect of KPA on the filamentation of *C. albicans*. YEM30 cells were diluted in the RPMI1640 medium and incubated with different doses of KPA at 37°C without shaking. KPA-free group was served as a negative control. Cells were imaged under a microscope in the bright field every 4 h. The bar indicates 50 μm.

Since hyphal development is the main pathogenic factor of *C. albicans*. We examined the effect of KPA on hyphal formation *in vitro* by microscopy. Figure 3 showed that KPA effectively inhibited the morphological transformation from yeast to the hyphae. YEM30 cells in 0 and 8 μg/mL groups were able to form regular hyphae within 3 h and complex networks of yeast within 12 h. 12 μg/mL of KPA inhibited the occurrence of hyphae within 12 h, while cells of 16 μg/mL KPA kept yeast form. This phenomenon suggested that KPA could inhibit the hyphal formation of *C. albicans*.

### KPA Suppressed the Biofilm Formation of *C. albicans*

**FIGURE 4:**
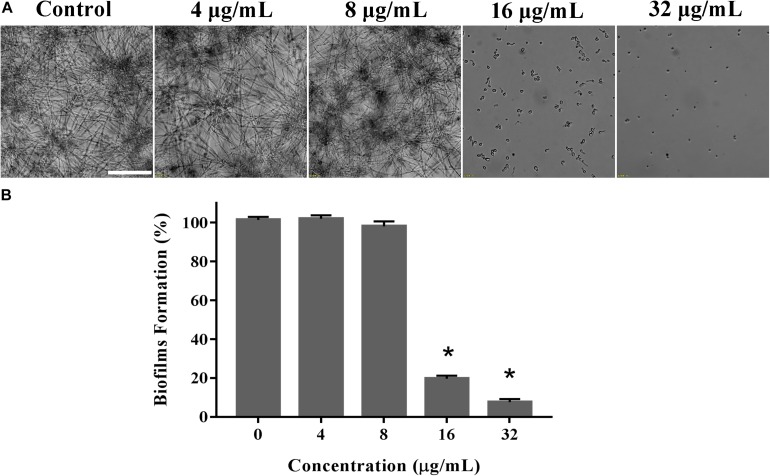
The inhibitory effect of KPA on biofilm formation of *C. albicans*. YEM30 cells were diluted in the RPMI1640 medium and incubated with different doses of KPA at 37°C without shaking for 24 h. A microscopic observation **(A)** and XTT reduction assay **(B)** were used to detect biofilm formation upon different treatment. The bar in panel **(A)** indicates 50 μm. Results in panel **(B)** are showed as means ± SDs. Asterisks (^∗^) represent significance with *P* < 0.01.

Biofilms confers intense drug resistance and the immune escape ability *C. albicans*, indicating that biofilm formation is an important virulence factor of *C. albicans* (Nett and Andes, 2006). Therefore, we qualitatively analyze the effect of KPA on the film formation microscopically. As shown in Figure 4A, KPA inhibited the biofilm formation completely at the dose of 16 μg/mL or higher, when only a few yeasts were observed at the bottom of the plate. This phenomenon was consistent with the results obtained from the XTT reduction assay (Figure 4B). When the concentration of KPA was increased 16 and 32 μg/mL, the inhibition rate of biofilms was 80 and 90%, respectively, suggesting that KPA could suppress biofilm formation by inhibiting the yeast-to-hyphal transition of *C. albicans*.

### cAMP Rescues the Inhibition Effect of KPA on Hyphae and Biofilm Formation

**FIGURE 5:**
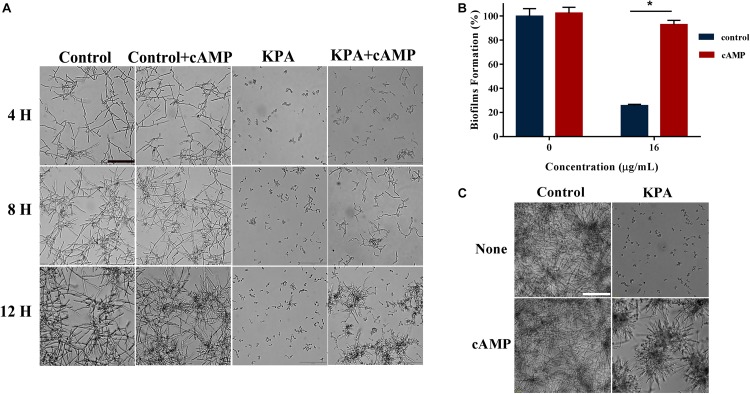
The effect of exogenous cAMP on hyphal and biofilm formation after KPA treatment. **(A)** Exogenous cAMP restored KPA-inhibited hyphal formation. YEM30 was diluted in RPMI1640 medium with or without 1 mM of db-cAMP and 12 μg/mL of KPA. After 4-hour incubation at 37°C, cells were imaged under a microscope in the bright field. **(B,C)** Exogenous cAMP partly restored KPA-inhibited biofilm formation. An XTT reduction assay **(B)** and microscopic observation **(C)** were used to detect the formed biofilms upon different treatment. The bars in panels **(A,C)** indicate 50 μm. Results in panel **(B)** are showed as means ± SDs. Asterisks (^∗^) represent significance with *P* < 0.01.

Cyclic adenosine monophosphate is the core molecule of regulatory network of morphological transformation, which can positively regulate the hyphal formation process of yeast cells (Sudbery, 2011). We conducted cAMP rescue experiment to observe whether cAMP affected the inhibitory effect of KPA on hyphae and biofilm formation. The results showed that the hyphae and biofilm growth inhibited by KPA (16 μg/mL) was restored by the addition of 1 mM exogenous db-cAMP within 12 and 24 h incubation (Figures 5A,C). The addition of db-cAMP restored the growth rate of yeast biofilms from 25.58 to 92.91% (Figure 5B). The restored effect of cAMP was continuous rather than transient. These results revealed that KPA could inhibit the growth of hyphae and biofilms by reducing the content of cAMP in yeast cells.

### KPA Stimulates Farnesol Secretion by Regulating Dpp3 Expression

**FIGURE 6:**
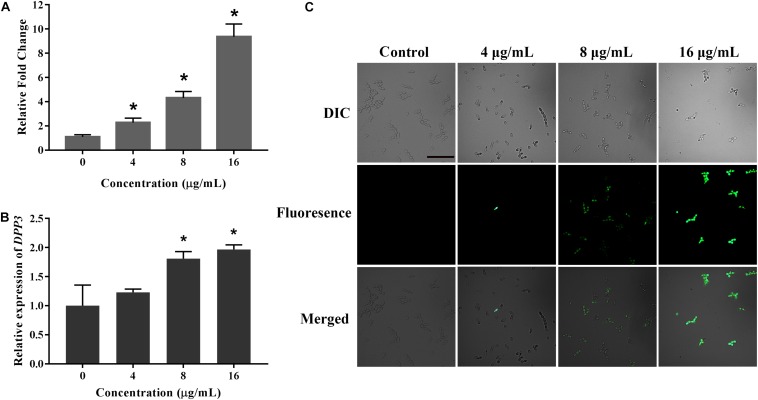
The effect of KPA on farnesol secretion and Dpp3 expression. **(A)** Farnesol was extracted from KPA cultured supernatants and quantified by GC-MS. **(B,C)** The expression of Dpp3 was examined by the fluorescence of BWP17-*DPP3*-*GFP* strain. Change in fluorescence was monitored by spectrofluorophotometry **(B)** and fluorescence microscopy **(C)**. The bar in panel **(A)** indicates 50 μm. Results in panels **(A,B)** are showed as means ± SDs. Asterisks (^∗^) in panels **(A,B)** represent significance with *P* < 0.05.

Farnesol, known as a quorum sensing molecule secreted by *C. albicans*, can inhibit the transition from yeast to hyphae. Therefore, we examined the effect of KPA on farnesol secretion. The result of GC-MS test results showed that KPA had a positive effect on the farnesol content of the supernatant. Compared with the control group, 4, 8, and 16 μg/mL of KPA treatment could increase the secretion of farnesol by 2.39, 4.16, and 9.15 times (Figure 6A).

Dpp3 is the key enzyme in the synthesis of farnesol (Navarathna et al., 2007). A Dpp3 labeled green fluorescent protein strain (BWP17-*DPP3*-*GFP*) was used for detecting the effect of KPA on Dpp3 expression. The results showed that the fluorescence intensity of Dpp3-gfp was positively correlated with the dose of KPA (Figure 6C). Similar to farnesol, KPA increased the DPP3 expression in a dose-dependent manner (Figure 6B). From the above results, we concluded that KPA could induce the synthesis of farnesol by stimulating the expression of *DPP3*, eventually inhibiting hyphal formation.

### KPA Prolonged the Survival Time of *C. albicans* Infected *C. elegans*

**FIGURE 7:**
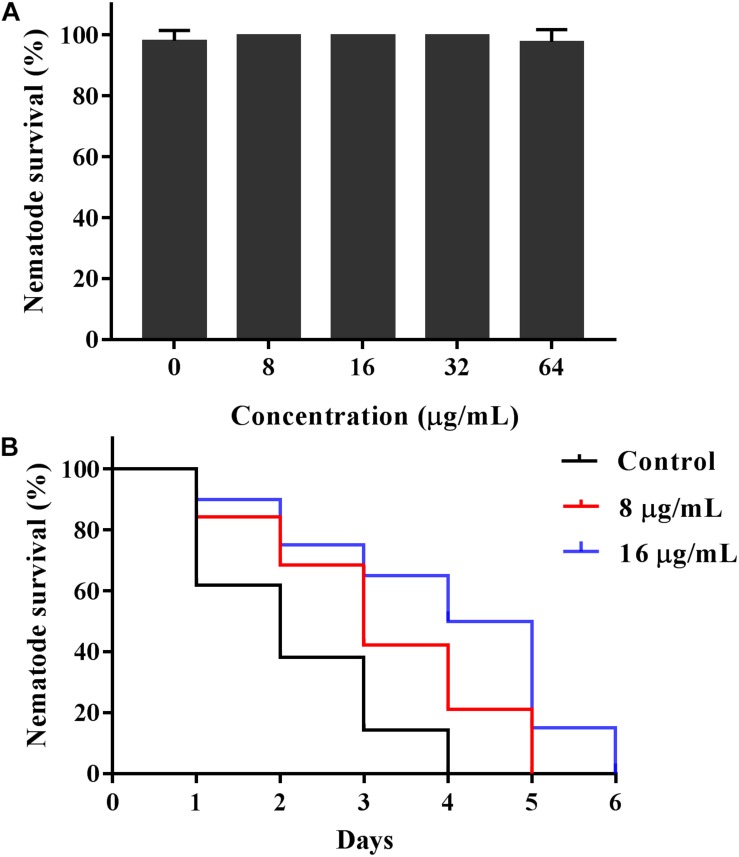
KPA increased the survival rate of *C. elegans* infected with *C. albicans*. **(A)** Healthy nematodes were incubated with or without KPA for 2 days. **(B)** Nematodes were infected with *C. albicans* YEM30 cells for 2 h and then moved to pathogen-free liquid medium in the presence of KPA or DMSO, nematodes infected with *C. albicans* in KPA-free group were served as a negative control.

Given the antifungal activity of KPA *in vitro*, we used the *C. elegans*-*C. albicans* infectious model to further investigate the antifungal activity of KPA *in vivo*. The survival curve showed that the nematode survival time of KPA treated group (8, 16 μg/mL) in the infectious model was longer than the control group (Figure 7B). In addition, the result of toxicity test indicated that high doses of KPA (4-fold of MIC) did not affect the survival of healthy wild type *C. elegans* within 2 days (Figure 7A). These data demonstrated the potential value of KPA for the treatment on *C. albicans* infection *in vivo*.

## Discussion

As an opportunistic pathogen, *C. albicans* can survive at several anatomically different sites, adapt to environment changes through morphological transformation and gradually form biofilms, causing fatal infections in immunodeficient patients (Noble et al., 2010). Morphological transformation is known as the most critical virulence factor inducing the biofilm formation in resistance to a variety of antifungal drugs including amphotericin B, acanthomycin and azole (Gulati and Nobile, 2016). The increasing emergence of drug resistance has increased the demand for new antifungal drugs and new antifungal strategies. It was found in this study that KPA had antifungal activity against *C. albicans*, and 1/2MIC effectively blocked the growth of yeast cells, indicating that low dose antifungal activity and high dose fungicidal activity of KPA. The results of MIC test showed that antifungal effect of KPA was independent of efflux regulatory proteins, including the ATP-binding cassette (ABC) transporter family members Cdr1 and Cdr2, and the major facilitator Mdr1. Efflux pump gene overexpression of is a vital reason for drug resistance (Cowen et al., 2015), suggesting KPA had a low probability to form resistance. In addition, the *C. elegans*–*C. albicans* infectious model showed that KPA protected *C. elegans* against infection through inhibiting yeast cells proliferation. More importantly, the low toxicity of KPA to healthy *C. elegans* increased the value of further research on KPA.

Hyphal formation is an amusing feature of *C. albicans*, which plays an important role in adhesion and biofilm formation (Modrzewska and Kurnatowski, 2015; Carradori et al., 2016). Given this characteristic, invasive hyphae are often detected in tissues of infected hosts. Correspondingly, hyphal defective *C. albicans* often exhibits lower virulence (Jabra-Rizk et al., 2016). Arresting or preventing morphogenesis means stopping infection. It was found in this study that KPA could reduce the virulence of *C. albicans* by inhibiting adhesion, yeast-to-hyphal transition and biofilm formation. These findings gave us further interest to explore its molecular mechanism.

Hyphal formation is influenced by various environmental factors such as quorum-sensing molecules (Sudbery, 2011). Farnesol, as a precursor of the isoprene/sterol pathway, is a quorum sensing molecule produced by microorganisms which is involved in preventing the morphological transformation and biofilm development of *C. albicans*, with cytotoxicity at a certain concentration (Shirtliff et al., 2009). *DPP3* is a gene encoding synthase of farnesol, and its expression was reported to affect the secretion of farnesol (Navarathna et al., 2007). The results demonstrated that KPA increased the secretion of farnesol by up-regulating Dpp3 expression. The development of hyphae is a complex process. Environmental factors are only external inducements of the morphological transformation, and regulation of the signal transduction pathway is believed to be the real internal cause (Sudbery, 2011). cAMP, synthesized by Cdc35, is considered as a key molecule in Ras1-cAMP-Efg1 pathway. It is a strong regulator for hyphal formation. Previous study has been proved that inhibition of cAMP synthesis could block yeast form growth of *C. albicans* under most hyphal induction conditions (Zhang et al., 2017). Furthermore, farnesol could regulate Ras-cAMP-Efg1 pathway by inhibiting the activity of Cdc35 to inhibit the development of hyphae (Lindsay et al., 2016). Ulterior exploration indicated that exogenous cAMP could restore the growth of hyphae and biofilms inhibited by KPA. The decrease of intracellular cAMP induced by KPA could directly inhibit filamentation of *C. albicans*.

In this study, we found that KPA could inhibit proliferation and various virulence factors of *C. albicans*, suggesting that KPA might be a promising antifungal agent to prevent Candida infection due to its low toxicity and resistance. Nevertheless, *in vivo* studies need to also be carried out to determine its biocompatibility, cytotoxicity, safety, and mode of action of KPA before it further used for biomedical applications.

## Data Availability Statement

All datasets generated for this study are included in the article/supplementary material.

## Author Contributions

HY participated in the extraction of KPA. HL and YL conceived the study and designed the experimental procedures. YL, MS, YW, and MY performed the experiments. YL, MS, HY, ZZ, and BG analyzed the data. ZZ, YL, and MS wrote the manuscript.

## Conflict of Interest

The authors declare that the research was conducted in the absence of any commercial or financial relationships that could be construed as a potential conflict of interest.

## Abbreviations

AMB: amphotericin B
cAMP: cyclic adenosine monophosphate
DMSO: dimethyl sulfoxide
GC-MS: gas chromatography-mass spectrometry
KPA: Kalopanaxsaponin A
MIC: minimal inhibitory concentration
SD: standard deviations.
